# sRNA expedites polycistronic mRNA decay in *Escherichia coli*

**DOI:** 10.3389/fmolb.2023.1097609

**Published:** 2023-03-03

**Authors:** Heung Jin Jeon, Yonho Lee, Monford Paul Abishek N, Changjo Kang, Heon M. Lim

**Affiliations:** ^1^ Department of Biological Sciences, College of Biological Sciences and Biotechnology, Chungnam National University, Daejeon, Republic of Korea; ^2^ Infection Control Convergence Research Center, College of Medicine, Chungnam National University, Daejeon, Republic of Korea

**Keywords:** sRNA, mRNA decay, RNase E, Spot42, galactose operon, polarity

## Abstract

In bacteria, most small RNA (sRNA) elicits RNase E-mediated target mRNA degradation by binding near the translation initiation site at the 5′ end of the target mRNA. Spot 42 is an sRNA that binds in the middle of the *gal* operon near the translation initiation site of *galK*, the third gene of four, but it is not clear whether this binding causes degradation of *gal* mRNA. In this study, we measured the decay rate of *gal* mRNA using Northern blot and found that Spot 42 binding caused degradation of only a specific group of *gal* mRNA that shares their 3′ end with full-length mRNA. The results showed that in the MG1655Δ*spf* strain in which the Spot 42 gene was removed, the half-life of each *gal* mRNA in the group increased by about 200% compared to the wild type. Since these mRNA species are intermediate mRNA molecules created by the decay process of the full-length *gal* mRNA, these results suggest that sRNA accelerates the mRNA decaying processes that normally operate, thus revealing an unprecedented role of sRNA in mRNA biology.

## 1 Introduction

In bacteria small RNAs (sRNA) regulates gene expression by base-pairing to specific regions of targeted mRNAs’ (Storz et al., 2011). The hexameric RNA chaperone, Hfq protein forms a nucleoprotein complex by binding the double-stranded region formed between sRNA and the target mRNA, recruiting RNaseE and facilitating mRNA degradation (Morita and Aiba, 2011; Prevost et al., 2011; De Lay et al., 2013; Updegrove et al., 2016; Kavita et al., 2018). Since most sRNA binding occurs where translation initiation occurs at the 5′ end of the target mRNA, sRNA binding creates a ribosome-free region onto which the RNase E-mediated transcript cleavage can take place. Subsequently, the generated RNA fragments are degraded by exoribonucleases. Thus, sRNA binding results in the degradation of the target mRNA (Masse and Gottesman, 2002; Masse et al., 2003; Morita and Aiba, 2011; Prevost et al., 2011; Kavita et al., 2018). Sometimes the transcription termination factor Rho binds onto the ribosome-free regions generated by sRNA binding and causes the termination of the target mRNA synthesis (Bossi et al., 2012; Sedlyarova et al., 2016; Chen et al., 2019).

**FIGURE 1:**
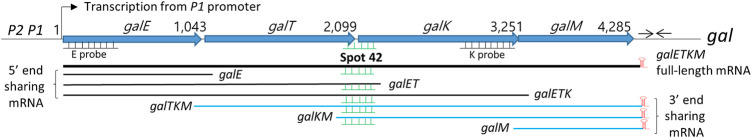
Map of *gal* operon and the 3′ and 5′end-sharing mRNA. *P1* and *P2* are the two promoters of the *gal* operon of *Escherichia coli* which are separated by five nucleotides. Numbers indicate *gal* nucleotide residue coordinates, where one is the start of transcription from the *P1* promoter. The number at the end of each gene belongs to the last nucleotide of the stop codon of that gene. Six nucleotides downstream of the stop codon of the *galM* gene is a 17-nucleotide inverted repeat sequence (head-to-head arrows) that forms the terminator hairpin (red) that terminates transcription and protects the full-length *galETKM* mRNA from 3′→5′ exonuclease digestion. Two DNA probes, E and K (500 bp) (underlines) used in the Northern blots are presented. The *galETKM* operon produces two kinds of mRNA groups: 3′ end-sharing and 5′ end-sharing. The 3′ end-sharing mRNA shares the same 3′ end with the full-length *galETKM* mRNA. The 5’end-sharing mRNA shares the same 5′ end with the *galETKM* mRNA. The E and K probes were used to detect the 5′end-sharing and 3′ end-sharing mRNA, respectively. Spot 42 (green) binds at the translation initiation region of the *galK* gene. Thus, Spot 42 binds to multiple mRNAs.

Spot 42 is a non-coding sRNA that is 109 nucleotides long. Unlike most sRNA, Spot 42 binds to the middle of polycistronic mRNA. More specifically, Spot 42 binds to the translation initiation region of the *galK* gene, which is the third of four genes in the *gal* operon (Figure 1) (Moller et al., 2002b). This binding causes two mutually exclusive events: 1) RNase E-mediated transcript cleavage; and 2) Rho-dependent transcription termination downstream from the Spot 42 binding site (Jeon et al., 2021). The 3′ ends of the transcripts generated by RNase E cleavage or transcription termination are processed to generate *galET* mRNA. Therefore, in contrast to most sRNA, the primary function of Spot 42 is to produce the target mRNA, which has a favorable impact on gene expression.

There are two groups of *gal* mRNA: 5′end-sharing and 3′end-sharing (Figure 1). Stochastic transcription termination by Rho at the end of each gene in the *gal* operon (*galE, galT, galK*, and *galM*) generates the mRNA species *galE*, *galET,* and *galETK*. These mRNA species share their 5′ ends with the full-length *galETKM* mRNA at their transcription initiation sites (Adhya, 2003; Lee et al., 2008; Wang et al., 2014). Thus, we termed this group “mRNA 5′end-sharing” (Jeon et al., 2020). Interestingly, RNase E-mediated transcript cleavage upstream of the translation initiation region of each *galETKM* cistron generates the mRNA species *galTKM*, *galKM*, and *galM* (Jeon et al., 2020). These mRNA species share their 3′ ends at the transcription termination site with *galETKM*. Thus, we termed this group “mRNA 3′end-sharing” (Jeon et al., 2020).

Since Spot 42 binds at the translation initiation site of the *galK* gene, Spot 42 binding may occur to *galETKM* mRNA as well as those of the 5′end-sharing (e.g., *galET*, *galETK*) and 3′end-sharing groups (e.g., *galTKM*, *galKM*) (Figure 1). In this study, we explored the possibility of Spot 42-mediated degradation in these two mRNA species groups. Based on our previous results that Spot 42 is involved in the degradation of *galKM* mRNA (Wang et al., 2015), we anticipated that Spot 42 binding on these *gal* mRNA species would also result in degradation. Interestingly, we found that Spot 42 only accelerated the decay rate of the *gal* mRNAs in the 3′end-sharing mRNA species group.

## 2 Materials and methods

### 2.1 Total RNA extraction

Total RNA was extracted from 2 × 10^8^ *Escherichia coli* cells grown at 37°C to an OD_600_ of 0.6 in LB media supplemented with the appropriate antibiotics and 0.5% galactose using the Direct-zol™ RNA MiniPrep kit (Zymo Research) as previously described (Wang et al., 2014; Jeon et al., 2020).

### 2.2 Northern blot analysis

The quantified total RNA (10 µg) was combined with the RNA loading buffer (×2) and 1 μg/mL ethidium bromide (EtBr) before being denatured for 10 min at 70°C. The samples were then resolved by gel electrophoresis for 2.5 h on a 1.2% (wt/vol) formaldehyde-agarose gel. Following electrophoresis, the RNA was transferred overnight using a downward transfer technique to a positively charged nylon membrane (Sambrook and Russell, 2006; Green and Sambrook, 2021) (Ambion, United States; TurboBlotter, Whatman, United Kingdom). Afterward, the nylon membrane was washed for 5 min with a ×2 SSC buffer before being baked at 80°C for 1 h. Northern blot E and K DNA probes (500 bp) were created *via* Polymerase chain reaction (PCR) amplification with primers corresponding to the *galE* region (from +27 to +527 in *gal* coordinates) and the *galK* region (from +2,103 to +2,604 in gal coordinates), which were subsequently radiolabeled with 32^P^ as previously described (Wang et al., 2014; Jeon et al., 2020). Next, the nylon membranes were hybridized per the manufacturer’s recommendations (Ambion, United States). After denaturing the DNA probe for 5 min at 95°C, 10 µL was added to the ULTRAhyb Ultrasensitive Hybridization solution (Invitrogen, United States) containing the pre-hybridized blot. The hybridization procedure was carried out overnight at 42°C. The blot was then washed twice in ×2 SSC and 0.1% SDS for 5 min at room temperature, followed by two 15-min washes in 0.2 × SSC and 0.1% SDS at 42°C. ImageJ software was used to calculate the relative intensity of the RNA bands (NIH). To determine the half-life of the mRNA, an XY scatter plot was created with the x-value representing time and the y-value representing the relative intensity of the RNA bands. An exponential decay curve was generated using the equation $y=a*\exp^{\left(-b*x\right)}$ , where *x* is time, and *a* and *b* are the *y*-intercept parameters respective to the relative band intensities at different time points. The half-life of each RNA band was determined using the equation $t1/2=\ln\left(2\right)/b=0.6931/b$ .

### 2.3 5′ Random amplification of cDNA ends (RACE)

The *E*. *coli* 5S rRNA 3′end was ligated to the 5′RNA ends during RNA preparation for the 5′end analysis of *gal* mRNA (Lee et al., 2008). Briefly, 2.5 µg of total RNA, 5 U T4 RNA ligase (Ambion, United States), and 10U rRNasin (Promega, United States) were ligated at 37°C for 3 h at a 15 µL reaction volume. Next, 1 µg of ligated RNA was reverse transcribed in a 20 µL reaction volume containing 4 U reverse transcriptase (Qiagen, Germany), 5 mM dNTP, 10 M random hexamer primer (Takara, Japan), and 10 U rRNasin for 2 h at 37°C. After that, RT-PCR was used to amplify the *gal* mRNA of interest. Briefly, 2 µL cDNA was used as a template for PCR amplification of the *gal* cDNA of interest at a total volume of 20 µL using 1 U of HotStar Taq DNA polymerase (Qiagen, Germany) with a forward primer complementary to the 3′end of the *E. coli* 5S rRNA, and a reverse primer specific to the *gal* cDNA of interest (Supplementary Table S1) (Lee et al., 2008). The 5′end of the *gal* cDNA was examined by extending the 32P-labelled DNA primer bound to a specific region of the amplified *gal* cDNA. The primer-extension reaction was carried out over 25 cycles at a volume of 20 μL, which included 10 µL amplified *gal* cDNA reaction, 1 µL 32^P^-labeled primer, 0.15 mM dNTP, and 1 U Taq polymerase. The extended-primer DNA was resolved on an 8% polyacrylamide-urea sequencing gel, with radioactive bands observed on the x-ray film. The number of nucleotides in the extended-primer DNA was used to determine the exact location of the 5′ends of the *gal* mRNA. The exact position of the 5′end of the *gal* mRNA in the nucleotide coordinates of the *gal* operon was determined by subtracting the number of nucleotides in the primer DNA from the number of nucleotides in the extended-primer DNA.

### 2.4 Reverse transcription-quantitative PCR (RT-qPCR)

Total RNA was extracted from 2 × 10^8^ MG1655, and MG1655hfq/pHfq *E. coli* bacteria cultivated at 37°C to an OD_600_ of 0.6 in LB medium supplemented with the appropriate antibiotics and 0.1% arabinose (Zymo Research) using the Direct-zol™ RNA MiniPrep kit. Turbo DNase I (Thermo Fisher Scientific, United States) was used to remove the genomic/plasmid DNA according to the manufacturer’s instructions. The reverse transcription reaction was performed as follows. 1 μg total RNA was incubated for 2 h at 37°C in a 20 μL reaction volume containing 4 U Omniscript reverse transcriptase (RT), ×10 RT buffer, 0.5 mM of each dNTP, 8 μM random hexamer primer, and 10 U rRNasin. RT-qPCR was performed in a 10 μL reaction volume containing 5 μL iQTM SYBR^®^ Green Supermix (Bio-Rad, United States), 3 μL RNase-free water, 0.5 μL each of 10 μM forward and reverse primers (QPCR_hfq-77-F and QPCR_hfq-184-R) (Supplementary Table S1), and 1 μL of the cDNA template. The following conditions were used: An initial denaturation step at 95°C for 3 min; 40 cycles of 10 s denaturation at 95°C; 20 s hybridization at 58°C; and 10 s of elongation at 72°C. The data from each sample was standardized using *rrsB*, which encodes 16S rRNA. The relative expression was calculated using the 2^−ΔΔCT^ method from the mean fold-changes of three replicates in three independent experiments (Livak and Schmittgen, 2001; Schmittgen, 2001).

## 3 Results

### 3.1 Spot 42 accelerates the mRNA decay rate of the 3′ end-sharing mRNA only

We measured the rate of decay of each *gal* mRNA species using wild type (WT) MG1655 and MG1655Δ*spf* cells in which the gene for Spot 42 was deleted from the MG1655 chromosome to see if Spot 42 is involved in the degradation of *gal* mRNA, (Wang et al., 2014). To do this, we added rifampicin at a final concentration of 100 μg/mL, the drug that inhibits transcription initiation, to the cells grown to an OD_600_ of 0.6 in LB. Aliquots were taken 2, 4, 6, and 8 min after drug addition. The total RNA was prepared from these cultures, then subjected to Northern blot analysis.

**FIGURE 2:**
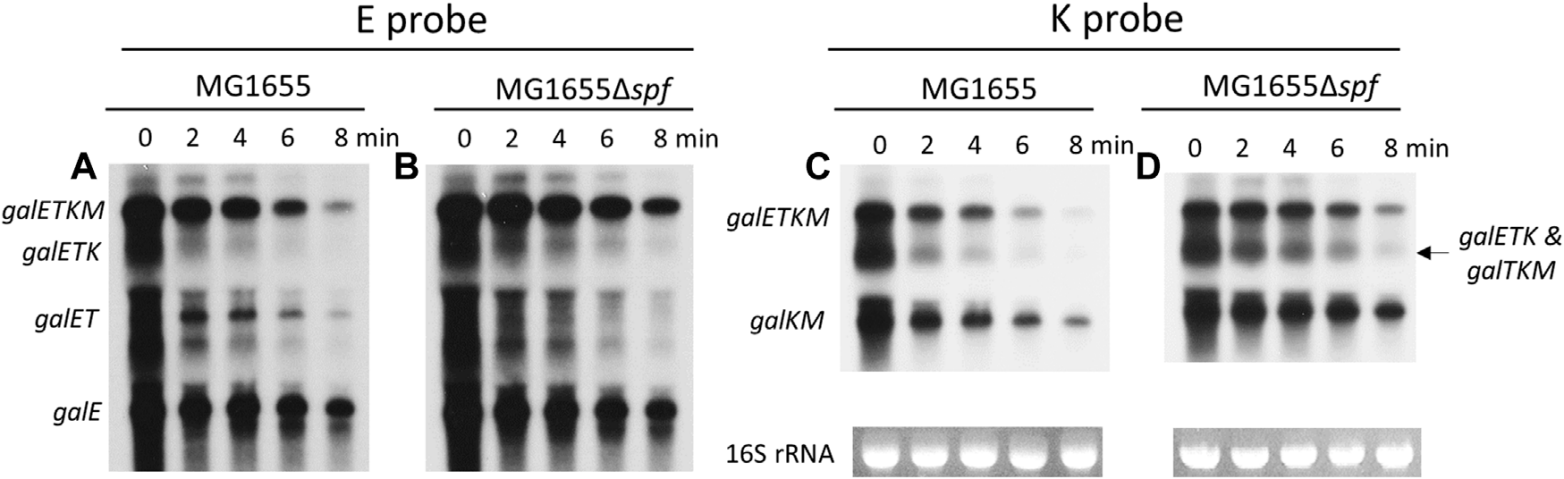
Spot 42 accelerates the mRNA decay rate of the 3′ end-sharing mRNA only. **(A, B)** Northern blots of *gal* mRNA from wild type (WT) MG1655 and MG1655Δ*spf* in the presence of rifampicin (100 μg/mL) with E probe. **(C, D)** Northern blots of *gal* mRNA from wild type (WT) MG1655 and MG1655Δ*spf* in the presence of rifampicin (100 μg/mL) with K probe.

First, the Northern blots were probed with the E probe that hybridizes to the first half of the *galE* gene (Figure 1). The E probe detects full-length and 5′ end-sharing mRNA species. These results showed that mRNA bands representing *galE*, *galET*, *galETK,* and *galETKM* decrease with increasing rifampicin treatment time (Figures 2A, B). Second, we measured the amount of each RNA band and calculated the half-life to measure the rate of decay (see Materials and Methods). The results showed that each of these mRNA species had a distinctive half-life. The full-length mRNA, *galETKM*, showed about 2.7 min half-life in the wild type (Table 1). Interestingly, the half-life of *galETKM* in MG1655Δ*spf* was measured to be 2.4 times longer (about 6.5 min) than that of *galETKM* in wild type (Table 1). The *galETK* mRNA showed a slight increase of 1.2 times in MG1655Δ*spf*. The half-life of *galET* was 2.3 min in wild type, but could not be measured in MG1655Δ*spf*, since *galET* mRNA is hardly produced in the absence of Spot 42 (Jeon et al., 2021). The half-life of *galE* mRNA was found to be the longest at 11.6 min in both strains (Table 1). These results suggest that Spot 42 is involved in the degradation of the full-length *galETKM* mRNA, but not in the degradation of the 5′ end-sharing mRNA.

**TABLE 1:** The half-life of the 5′-sharing mRNA. These were measured from the E-probed blots. The values are the average of three independent experiments.

	WT	Δspf	Δspf/WT
*galETKM*	2.69 ± 0.36	6.44 ± 0.96	2.4
*galETK*	1.78 ± 0.16	2.33 ± 0.16	1.2
*galET*	2.31 ± 0.18	—	—
*galE*	11.61 ± 0.90	11.87 ± 0.55	1.0

The same blots were washed, then re-probed using the K probe, which binds to the second half of the *galK* gene. The K probe detects the full-length and the 3′ end-sharing *gal* mRNA species (*galTKM*, *galKM*). It should be noted that the K probe also detects one of the 5′ end-sharing mRNA (*galETK*). Thus, in the K-probed Northern blots, the RNA band size of 3.2 kb represents both *galTKM* (3′ end-sharing) and *galETK* (5′-end sharing) mRNA. The K-probed Northern blots showed that mRNA bands representing *galETKM*, *galTKM*, *galETK*, and *galTKM* decreased with increasing rifampicin treatment time (Figures 2C, D). The half-life of the full-length mRNA (*galETKM*) was the same as in the E-probed blots: The half-life of *galETKM* increased 2.4 times compared to MG1655Δ*spf* (Table 2). The mixed half-life of *galTKM/galETK* in MG1655Δ*spf* was 1.7 times that of wild type (Table 2). Since the half-life of *galETK* increased 1.2 times, that of the mixed *galETK/galTKM* increased 1.7 times, which suggests that Spot 42 works on the 3′ end-sharing *galTKM* only. The half-life of *galKM* was 5.5 min in wild type, but 11.8 min in MG1655Δ*spf*; that is, the half-life of *galKM* was 2.1 times longer in MG1655Δ*spf* (Table 2). Thus, we found that Spot 42 accelerated the decay rate of *galETKM* and the 3′ end-sharing mRNA (*galTKM* and *galKM*).

**TABLE 2:** The half-life of the 3′-sharing mRNA. These were measured from the K-probed blots. The values are the average of three independent experiments.

	WT	Δspf	Δspf/WT
*galETKM*	2.69 ± 0.36	6.44 ± 0.96	2.4
*galTKM/galETK*	1.95 ± 0.19	3.28 ± 0.63	1.7
*galKM*	5.55 ± 0.31	11.87 ± 1.70	2.1

Since Spot 42 binds to the middle of the *gal* operon in the *galT-galK* cistron junction region (Figure 1), the results from the half-life measurements of the *gal* mRNA in wild type MG1655 and MG1655Δ*spf* strains demonstrate that Spot 42 enhances the rate of decay of the 3′ end-sharing mRNA of the *gal* mRNA species in wild type. In the wild type, Spot 42 promoted the decay rate of the full-length mRNA *galETKM* and the 3′ end-sharing mRNA (*galTKM*, *galKM*). This promotion effect could be up as much as 200% during the exponential growth period.

### 3.2 Region III of spot 42 is involved in mRNA degradation of the 3′ end-sharing mRNA only

**FIGURE 3:**
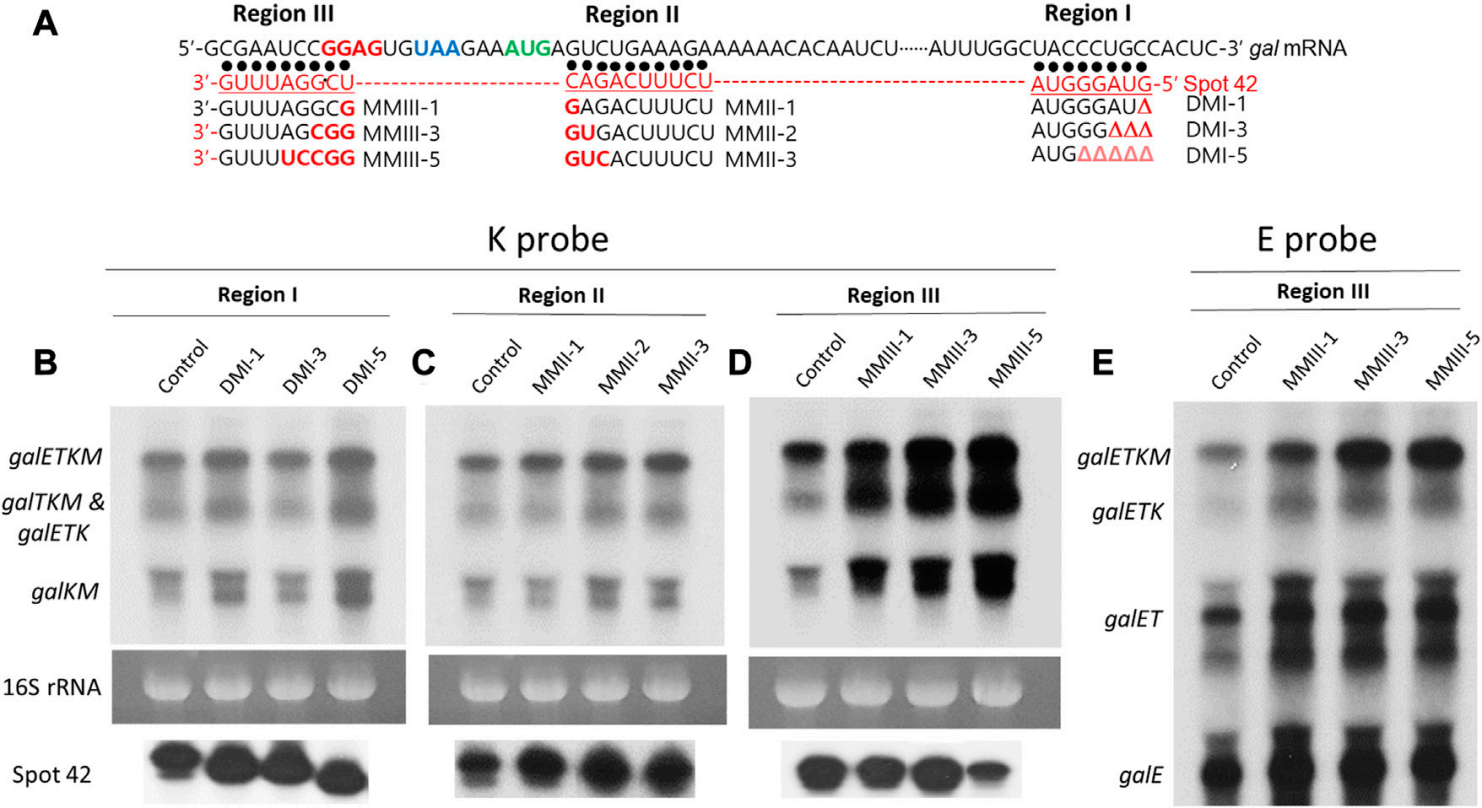
Region III of Spot 42 is involved in mRNA degradation of the 3′ end-sharing mRNA only. **(A)** Nucleotide sequence of *gal,* Spot 42, and Spot 42 mutants. The Shine and Dalgarno sequence for translation initiation of *galK* is presented in red. The translation stop codon of *galT* and the translation initiation codon of *galK* are presented in blue and green, respectively. Also, about 50 nucleotide sequences of the 5′ portion of Spot 42 are presented in red below the *gal* sequence. The three regions of Spot 42 that form perfect base pairings with the *gal* nucleotide sequences are identified as regions I, II, and III. Black dots represent base pairing. Spot 42 mutations in each region are listed under the corresponding region by name. Nucleotide changes are marked in red. The region I mutations are nucleotide deletions denoted as Δ in red. **(B)** K-probed Northern blot of the region I mutant; **(C)** K-probed Northern blot of the region II mutant; **(D)** K-probed Northern blot of the region III mutants; **(E)** E-probed Northern blot of the region III mutants.

Three separate regions (regions I, II, and III) of about 10 nucleotides each are found within the 50 nucleotides of the 5′ portion of Spot 42 (Supplementary Figure S1). These regions allow for perfect base-pairing with their corresponding regions of *gal* mRNA at the *galT-galK* cistron junction (Figure 3A). In our previous study, we showed that the base-pairings of regions I and II result in the generation of *galET* mRNA, but that region III base-pairing plays no role in the generation of *galET* mRNA (Jeon et al., 2021). From these results, we suggested that the effect of Spot 42 could differ, depending on which portion of Spot 42 base pairs to the corresponding target region of mRNA. We anticipated that the effect of region III base-pairing of Spot 42 caused the enhancement of the mRNA decay rates shown in Figure 2.

We used a series of substitution or deletion mutations in regions I, II, and III of Spot 42 to determine which region is involved in the decay of *galETKM, galTKM*, and *galKM* observed in Figure 2, (Figure 3A) (Jeon et al., 2021). The necessary Spot 42 mutations were generated in a pBR322-derived plasmid (pSpot42) in which the Spot 42 gene was cloned under the *lac* promoter (Beisel and Storz, 2011). The Spot 42 mutants were assayed in MG1655Δ*spf* in pSpot42.

MG1655Δ*spf*/pSpot42 cells were grown to an OD_600_ of 0.6 in LB. From these cultures, the total RNA was isolated and subjected to Northern blot analysis that was subsequently probed with the K probe. The Spot 42 concentrations were measured in the MG1655Δ*spf*/pSpot42 cells harboring each Spot 42 mutant by 5′ RACE assay to assess the amount of 5′ ends of the RNA of interest *in vivo* (see Matrials and methods), then were presented below each lane of the Northern blots (Figure 3). The concentration of each Spot 42 mutant was more or less the same as that found in wild type Spot42. The mutant additions to regions I (Figure 3B) and II (Figure 3C) showed no noticeable effect on the amount of *galETKM*, *galTKM,* and *galKM*, except for mutant DM1-5 (five nucleotides deleted from the 5′ end of Spot 42), which caused an about 30% increase compared to the rest of the mRNA in the Northern blot (Figure 3B). The Spot 42 mutations in region III resulted in an increased amount of *galETKM*, *galTKM,* and *galKM* since the Spot 42 mutant harbored more mispaired nucleotides (Figure 3D). The single substitution mutants (Spot42MMIII-1), triple substitution mutants (Spot42MMIII-3), and quintuple substitution mutants (Spot42MMIII-5) in region III increased by 30%, 70%, and 90% in *galETKM*, by 50%, 90%, and 100% in *galTKM,* and by 60%, 100%, and 150% in *galKM* (Figure 3D), respectively(Figure 3D).

To determine the effect of the region III mutants on the 5′-sharing mRNA, we stripped off the K probe and re-probed the blot with the E probe. The findings revealed a modest rise in all 5′end-sharing mRNA, but no discernible variations in their levels were found (Figure 3E). The full-length *galETKM* showed the same increasing trend as in the K-probed blot in Figure 3D (Figure 3E). It is particularly interesting to see that *galETK*, a 5′end-sharing mRNA, was not increased in all region III mutants. These results corroborated the results that the region III base pairing of Spot 42 is only involved in the degradation of *galETKM*, and the 3′ end-sharing mRNA (*galTKM* and *galKM*).

### 3.3 Spot 42-mediated gal mRNA degradation needs Hfq

Hfq protein is known to be involved in sRNA-mediated mRNA degradation (Moller et al., 2002a; Morita et al., 2005; Morita and Aiba, 2011; Prevost et al., 2011; Kavita et al., 2018). To determine whether Hfq is involved in the Spot 42-mediated 3′end-sharing mRNA degradation, we created MG1655Δ*hfq*, a strain in which the *Hfq* gene was deleted from the MG1655 chromosome. We also cloned the *Hfq* gene in a plasmid pBAD (Guzman et al., 1995) and placed the *Hfq* gene under the control of the P_*BAD*_ promoter. This created an Hfq-producing plasmid we referred to as pHfq. MG1655Δ*hfq* and MG1655Δ*hfq* harboring pHfq were grown to an OD_600_ of 0.6 in LB with 0.1% arabinose. The total RNA from these cultures was subjected to a Northern blot, then subsequently probed with the K probe.

**FIGURE 4:**
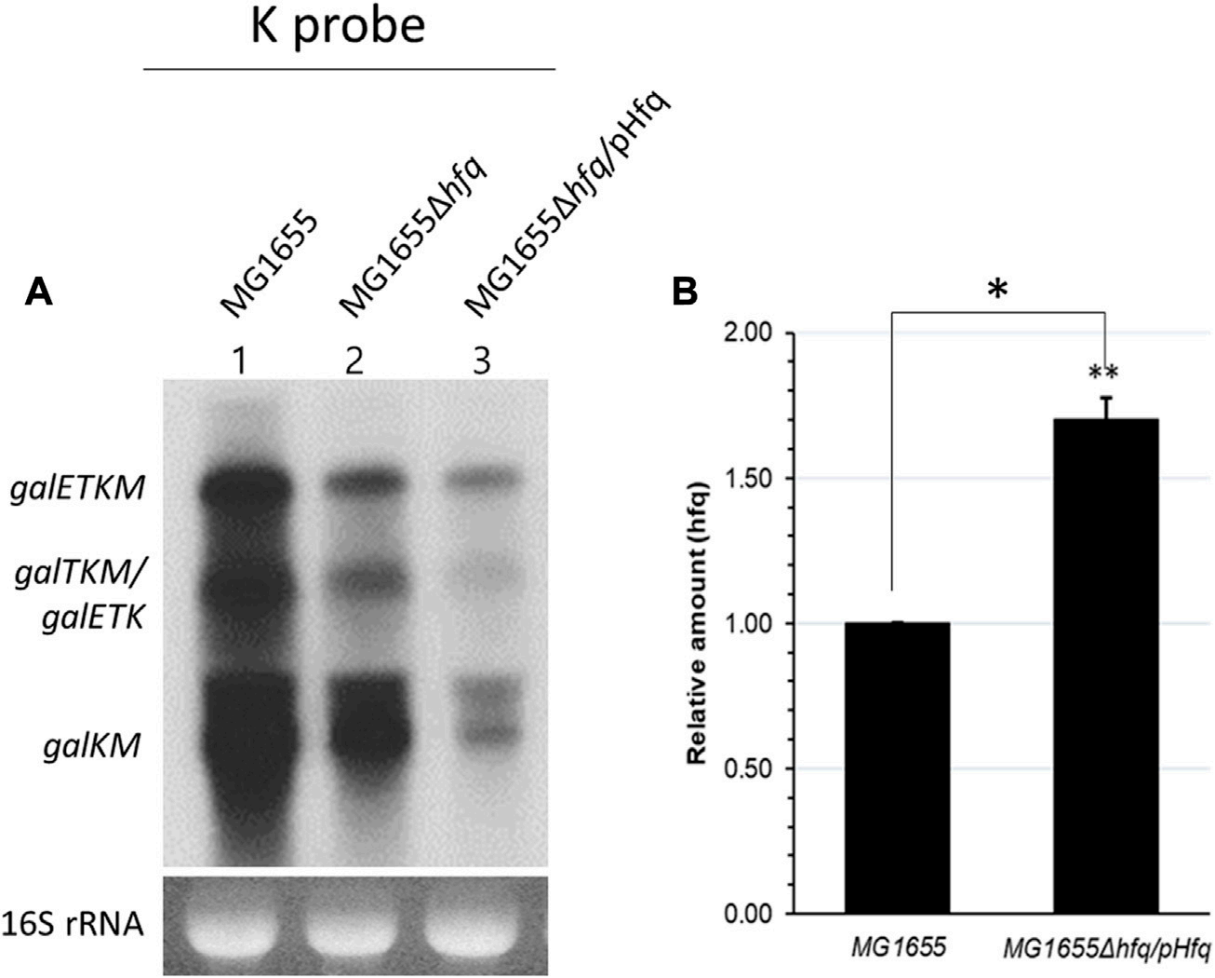
Spot 42-mediated *gal* mRNA degradation needs Hfq. **(A)** K-probed Northern blot of MG1655 (lane 1), MG1655Δ*hfq* (lane 2), and MG1655Δ*hfq* harboring the Hfq expression plasmid pHfq (lane 3). **(B)** The relative amount of *hfq* mRNA was measured by using qRT-PCR in MG1655 and MG1655Δhfq harboring pHfq plasmid. Error bars represent the mean fold-change ±standard deviation from three independent experiments. *p-value ≤ 0.05 (statistically significant).

Unexpectedly, the K-probed Northern blot showed that the amount of *galETKM* and the 3′end-sharing mRNA (also *galETK*) in the MG1655Δ*hfq* strain decreased to about 50% of wild type (lane 2; Figure 4A). However, we reasoned that the presence of the Hfq protein in MG1655Δ*hfq* would further lower the amount of the 3’end-sharing mRNA if Hfq is a needed factor for the observed degradation. It is unclear why the production of *gal* mRNA decreased in wild type cells in the absence of Hfq. The quantities of *galETKM*, *galTKM*/*galETK*, and *galKM* in MG1655Δ*hfq*/pHfq were 50%, 30%, and 35%, respectively, lower than they were in MG1655Δ*hfq* (lane 3, Figure 4A). Quantitative RT-PCR measurement of *Hfq* mRNA showed that *Hfq* expression increased by 70% in MG1655Δ*hfq*/pHfq compared to MG1655 (Figure 4B), suggesting that Hfq protein increased accordingly. Thus, Hfq is needed for the Spot 42-mediated mRNA degradation of *galETKM* and the 3′ end-sharing mRNA (*galTKM* and *galKM*).

### 3.4 Spot 42 negatively regulates RNase E cleavage that generates galKM

**FIGURE 5:**
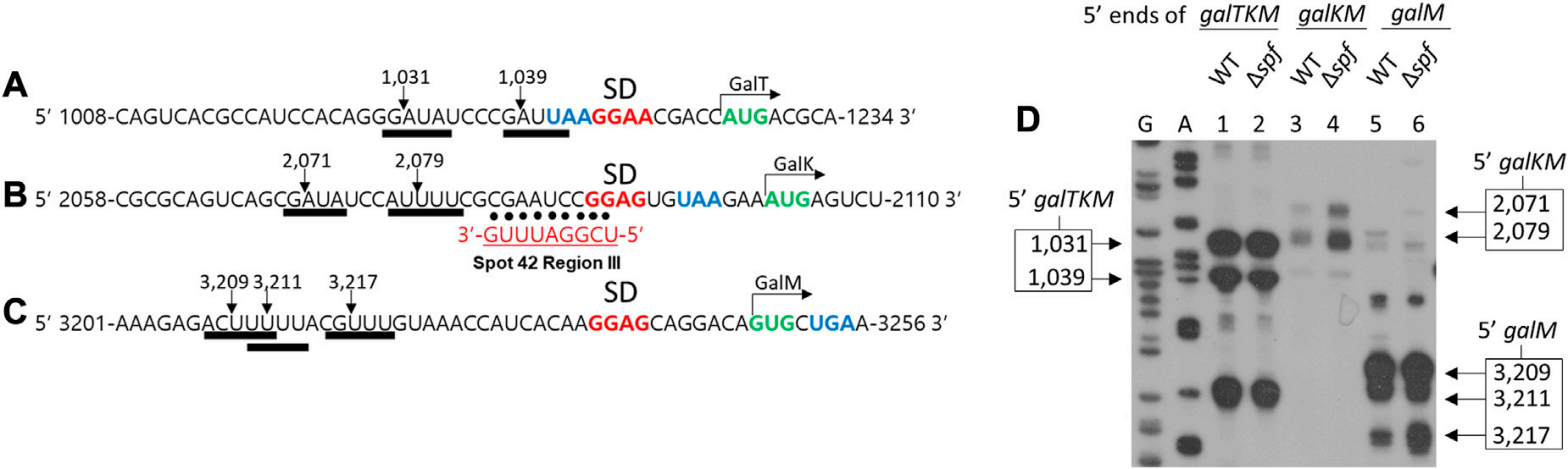
Spot 42 negatively regulates RNase E cleavage that generates *galKM.* Nucleotide sequences upstream of the translation initiation codons of *galT* **(A)**, *galK* **(B)**, and *galM* **(C)**, show the correspondence of the cleavage positions (inverted arrows) of the RNase E consensus cleavage sites (thick underlines). Also highlighted are the putative Shine–Dalgarno sequences (in bold red) and the initiation (green) and termination (blue) codons. The RNase E cleavages in **(A)**, **(B)**, and **(C)** result in the generation of the 5′ end of *galTKM*, *galKM,* and *galM*, respectively. Base pairing of region III to the corresponding regions of *gal* are presented with dots. **(D)** 5′RACE results show the 5′ ends of *galTKM* at 1,031 and 1,039 in wild type (WT) (lane 1) and Δ*spf* (lane 2), the 5′ ends of *galKM* at 2,071 and 2,079 in wild type (WT) (lane 3), and Δ*spf* (lane 4), and the 5′ ends of *galM* at 3,209, 3,211, and 3,217 in wild type (WT) (lane 5) and Δ*spf* (lane 6).

RNase E cleaves several nucleotides upstream of the translation initiation sites of *galT*, *galK,* and *galM* genes in the full-length mRNA *galETKM*, and generates the 5′ end of the 3′ end-sharing mRNA species *galTKM*, *galKM*, and *galM*, respectively (Figures 5A–C) (Jeon et al., 2020). Interestingly, region III of Spot 42 perfectly base pairs with the *gal* mRNA region several nucleotides downstream from the RNase E cleavage sites at the translation initiation region of the *galK* gene (Figure 5B). The proximity of the RNase E cleavage and the region III binding sites suggest that Spot 42 binding could interfere with the RNase E cleavage at 2,079 and 2,071 (Figure 5B), which are the *gal* nucleotide residue coordinates.

To test the effect of Spot 42 binding on RNase E cleavage, we assayed the 5′ end of the 3′ end-sharing mRNA species. Using the 5′RACE assay, we quantified and located their 5′ ends in the presence and absence of Spot 42. To do this, wild type MG1655 and MG1655Δ*spf* cells were grown to an OD_600_ of 0.6 in LB and total RNA from the cultures was subjected to a 5′RACE assay. The results confirmed that, in wild type cells, the 5′ ends of *galTKM* were at two sites (1,031 and 1,039), those of *galKM* were also at two sites (2,071 and 2,079), and those of *galM* were at three sites (3,209, 3,211, and 3,217) in lanes 1, 3, and 5, respectively (Figure 5D). In MG1655Δ*spf*, the 5′ ends of *galTKM* and *galM* were formed at the same sites with the same amounts. The two 5′ ends of *galKM* increased in MG1655Δ*spf*. The amount of both 5′ ends of *galKM* at 2,071 and 2,079 was increased by 190% and 260% compared to wild type, respectively, in MG1655Δ*spf* (lane four in Figure 5D). These results showed that Spot 42 inhibits RNase E cleavage upstream of the *galK* translation initiation site. Since this RNase E cleavage generates *galKM* mRNA (Jeon et al., 2020), Spot 42 binding inhibited the production of *galKM* mRNA, possibly through its region III base pairing. Thus, Spot 42 downregulates *galKM* mRNA production by accelerating decay and inhibiting the generation of *galKM* mRNA.

## 4 Discussion

### 4.1 Spot 42 expedites the decay process of the full-length mRNA of gal

**FIGURE 6:**
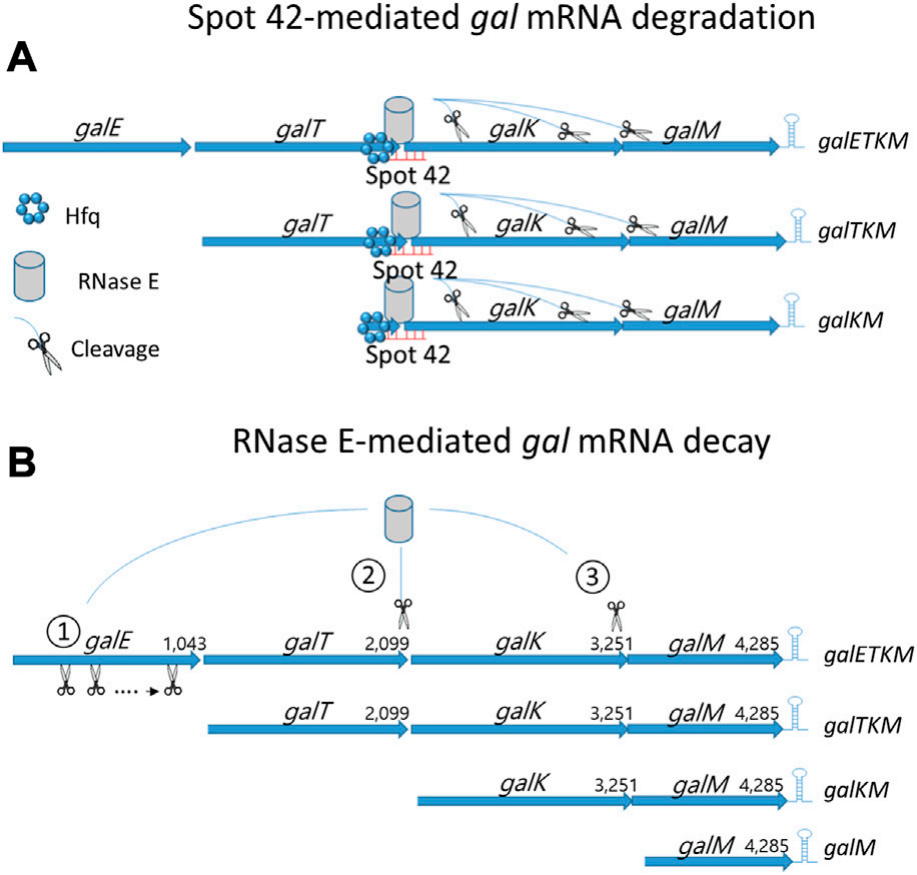
mRNA decay of the multicistronic *galETKM.* **(A)** Spot 42-mediated *gal* mRNA degradation: Hfq-mediated region III base pairing to *galETKM*, *galTKM,* and *galKM* recruit RNase E and subsequently induce RNase E cleavage downstream. **(B)** RNase E-mediated *gal* mRNA decay: 1) Generation of *galTKM* mRNA*:* The first gene of the *gal* operon, *galE*, is removed by progressive and successive RNase E cleavages initiated at the 5′ end of *galETKM* mRNA. This progressive RNase E cleavage is followed by the 3→5′ exo-ribonucleolytic digestion of the released RNA fragment stops at the translation initiation site of the next *galT* gene, generating *galTKM* mRNA. The sites of RNase E cleavage that generate the 5′ ends of *galTKM* are presented in Figure 5A. The 5′ ends are experimentally shown (lane 1; Figure 5D). 2) Generation of *galKM* mRNA: Sometimes RNase E cleaves in the middle of *galETKM* mRNA, several nucleotides upstream from the translation initiation site of the *galK* gene, followed by the 3→5′ exo-ribonucleolytic digestion of the 5′ portion RNA fragment, which generates *galKM* mRNA, *galKM*. The sites of the RNase E cleavages that generate the 5′ ends of *galKM* are presented in Figure 5A. The 5′ ends are experimentally shown (lane 3; Figure 5D). 3) Generation of *galM* mRNA: RNase E cleavages on the upstream sites from the translation initiation of the last gene of the operon, *galM*, followed by the 3→5′ exo-ribonucleolytic digestion of the released RNA fragment generates *galM* mRNA, *galM*. The sites of the RNase E cleavages that generate the 5′ ends of *galM* are presented in Figure 5A. The 5′ ends are experimentally shown (lane 5; Figure 5D). RNase E also cleaves a few nucleotides upstream from the transcription terminator hairpin at the 3′ end of *galM* mRNA. Exoribonucleases rapidly remove the 5′ portion of the *galM* mRNA upstream of the hairpin structure, completing the decay process of the multicistronic full-length mRNA down to the nucleotides. Thus, the decay of the full-length *galETKM* mRNA proceeds by generating the 3′ end-sharing mRNA (*galTKM*, *galKM,* and *galM*), which we defined as ‘decay intermediates’ (Jeon et al., 2020).

The Hfq protein promotes sRNA binding to target mRNA (Moller et al., 2002a; Waters and Storz, 2009; De Lay et al., 2013; Updegrove et al., 2016; Kavita et al., 2018) and recruits RNase E for target mRNA degradation (Aiba, 2007; Morita and Aiba, 2011; De Lay et al., 2013; Kavita et al., 2018). In this study, we showed that Hfq is involved in the Spot 42-mediated mRNA degradation of *gal* mRNA belonging to the 3′end-sharing group only (Figure 4). With this information, we propose the following model: Region III base pairing with Hfq on *galETKM*, *galTKM,* and *galKM* recruits RNase E, thus causing RNase E-mediated progressive RNA cleavages downstream from the binding sites (Figure 6A). Possibly, 3’→5′ exo-ribonucleolytic digestion of the released RNA fragments could result in the degradation of *gal* mRNA in *galETKM*, *galTKM,* and *galKM* (Figure 6A).

Most mRNA decay in *E. coli* proceeds with an initial RNase E cleavage of mRNA followed by the exo-ribonucleolytic digestion in the 3’→5′ direction of the 5′ portion of the mRNA (Belasco et al., 1985; Mott et al., 1985; Hui et al., 2014; Wang et al., 2019). RNase E is an endoribonuclease with a higher affinity to the mono-phosphorylated 5′ end of mRNA (Mackie, 1998; Jiang and Belasco, 2004; Callaghan et al., 2005). Thus, the initial RNase E cleavage could occur at the 5′ end of mRNA (5′-end dependent), or it could sometimes occur at the middle region of mRNA (Direct entry) (Clarke et al., 2014). In our previous report of Jeon et al. (2020), we found that RNase E cleavage can occur a few nucleotides upstream from the translation initiation site of each cistron of *galETKM* mRNA by employing both modes of the initial RNase E cleavages. The 5′ portion of mRNA is rapidly removed by exo-ribonucleolytic digestion. Jeon et al. (Jeon et al., 2020) proposed that the decay of the full-length *gal* mRNA (*galETKM*) proceeds by generating intermediate mRNA species with fewer but fuller cistrons (i.e., the 3′end-sharing mRNA; *galTKM*, *galKM*, and *galM*) (Figure 6B).

Taking these results together, we suggest that there are two layers of *gal* mRNA decay in *E. coli*: RNase E-mediated *gal* mRNA decay (Figure 6B) and Spot 42-mediated *gal* mRNA degradation (Figure 6A). Based on the results from this study, we propose that the Spot 42-mediated mRNA degradation is the molecular mechanism causing the Spot 42-accelerated decay rate of the 3′end-sharing mRNA (Tables 1, 2). Based on this reasoning, we conclude that Spot 42 accelerates the normal decaying process of the full-length mRNA in the cell.

### 4.2 Spot 42 elicits multiple outcomes

Spot 42 binding to *gal* mRNA causes multiple outcomes. Interestingly, these outcomes depend on which region Spot 42 base pairs within the corresponding *gal* mRNA. The base pairing of region I cause the generation of *galET-long* mRNA (Jeon et al., 2021), And Region II base-pairing causes the generation of the *galET-short* mRNA that is about 30 nucleotides shorter than *galET-long* in the 3′ end (Jeon et al., 2021). These two outcomes ended up producing *galET* mRNA that appears as a single band of 2.2 kb in the Northern blot (Jeon et al., 2021). This study demonstrated that the region III base-pairing causes the degradation of the full-length *galETKM* and the 3′ end-sharing mRNA (*galTKM* and *galKM*).

Spot 42 binds to the sole binding site in the *gal* operon at the cistronic junction between the *galT* and *galK* genes. The translation stop codon of *galT* is three nucleotides upstream from the translation initiation codon of *galK*, and a putative Shine–Dalgarno sequence for *galK* resides two nucleotides upstream of the *galT* stop codon (Figure 3A). Thus, the translation initiation of the next gene, *galK*, immediately follows the translation termination of the preceding gene, *galT*. Our finding that Spot 42 binding to *gal* mRNA causes different outcomes should be understood to concern the translational activity of the ribosome at the cistron junction during Spot 42 binding. Translation of the lead ribosome is physically (Burmann et al., 2010; Proshkin et al., 2010) and functionally (Svetlov and Nudler, 2012; Wang et al., 2020) coupled with transcription in *E. coli*. Thus, for example, it has been suggested that region II base-pairing blocks the translation initiation activity of *galK* in the lead ribosome, causing Rho-dependent transcription termination that leads to the generation of *galET-short* mRNA (Jeon et al., 2021). We also suggest that region I base-pairing blocks the translation initiation activity of *galK via* the ordinary ribosome, causing a ribosome-free region downstream and, in turn, inducing RNase E cleavage that leads to the generation of *galET-long* mRNA (Jeon et al., 2021).

If Spot 42 binding would have interfered with the translation termination of *galT*, it would have ended up producing incomplete GalT proteins. Thus, theoretically, Spot 42 binding should not prevent the translation termination of *galT*. Based on these arguments, we propose that region III base pairing occurs when there is no translation termination of *galT*. that is, region III base pairing to *galETKM* or *galTKM* mRNA should occur when the gene(s) upstream of region III base pairs are free from translating ribosomes. In addition, region III base pairing to *galETKM* or *galTKM* mRNA and *galKM* would result in the removal of ribosomes from the downstream genes. Thus, region III base pairing occurs and causes ribosome-free, non-translating mRNA that needs to be removed.

The multiple outcomes from single Spot 42 binding are possibly caused by Spot 42 binding to the middle of multicistronic mRNA at different times. We propose that if Spot 42 binding occurs on the transcript mRNA during transcription (i.e., in-transcriptionally), it should lead to the generation of *galET* mRNA. If, however, Spot 42 binding occurs to mRNA produced after transcription termination (i.e., post-transcriptionally), it should lead to degradation. Perhaps the same phenomena would occur to any sRNA that binds to the middle of a multicistronic mRNA. For example, it has been reported that sRNA RyhB binding to the translation initiation site of the second gene of the polycistronic mRNA *iscRSUA* causes the degradation of *iscRSUA* mRNA (Desnoyers et al., 2009).

### 4.3 The physiological role of spot 42

Overall, Spot 42 binding accelerates the decay rate of the 3′ end-sharing mRNA and at the same time inhibits the generation of *galKM* mRNA (Figure 5) (Wang et al., 2015), which harbors *galK* and *galM,* the last two genes of the operon. Importantly, Spot 42 binding generates *galET* mRNA (Jeon et al., 2021), which harbors *galE* and *galT*, the first two genes of the operon. Thus, these effects lead to the downregulation of the *galT*-downstream expression of these genes; thereby escalating the polarity of *gal* gene expression already established by the Rho-dependent transcription termination at the end of each gene in the operon (Wang et al., 2015). Since cAMP-CRP downregulates Spot 42 transcription (Beisel and Storz, 2011), escalating or de-escalating the polarity in *gal* gene expression could depend on cAMP concentrations, with less polarity resulting in higher cAMP concentrations as previously demonstrated (Ullmann et al., 1979; Wang et al., 2015).

## Data Availability

The original contributions presented in the study are included in the article/Supplementary Materials, further inquiries can be directed to the corresponding authors.
