# Supplementary material for: sRNA expedites polycistronic mRNA decay in Escherichia coli
Source: Front Mol Biosci. 2023 Mar 3;10:1097609. doi: 10.3389/fmolb.2023.1097609 (PMC10020718; doi:10.3389/fmolb.2023.1097609)
Supplement: Supplementary file 1 [file DataSheet1.PDF]

Supplementary Material

sRNA expedites polycistronic mRNA decay in *Escherichia coli*

Heung Jin Jeon[1,2*], Yonho Lee[1], Monford Paul Abishek N[1], Changjo Kang[1], Heon M. Lim[1*]

[1] Department of Biological Sciences, College of Biological Sciences and Biotechnology, Chungnam National University, Daejeon, Republic of Korea

[2] Infection Control Convergence Research Center, College of Medicine, Chungnam National University, Daejeon, Republic of Korea

Running Title: sRNA accelerates mRNA decay

*To whom correspondence should be addressed.

Email: livinglogos@cnu.ac.kr (H.J.J), and hmlim@cnu.ac.kr (H.M.L).

Tel: +82-42-280-6980 (H.J.J), and +82-42-821-6276 (H.M.L).

This PDF file includes:

S1 Figure

S1 Table

References

1 **Supplementary Figure S1. Secondary structure prediction of the full-length Spot42 RNA performed by RNA fold.** Spot 42 base-pairing regions (Region I, II, and III) with *gal* mRNA are shown. Region I: 1-8 nt, Region II: 27-36 nt and Region III: 53-61 nt.

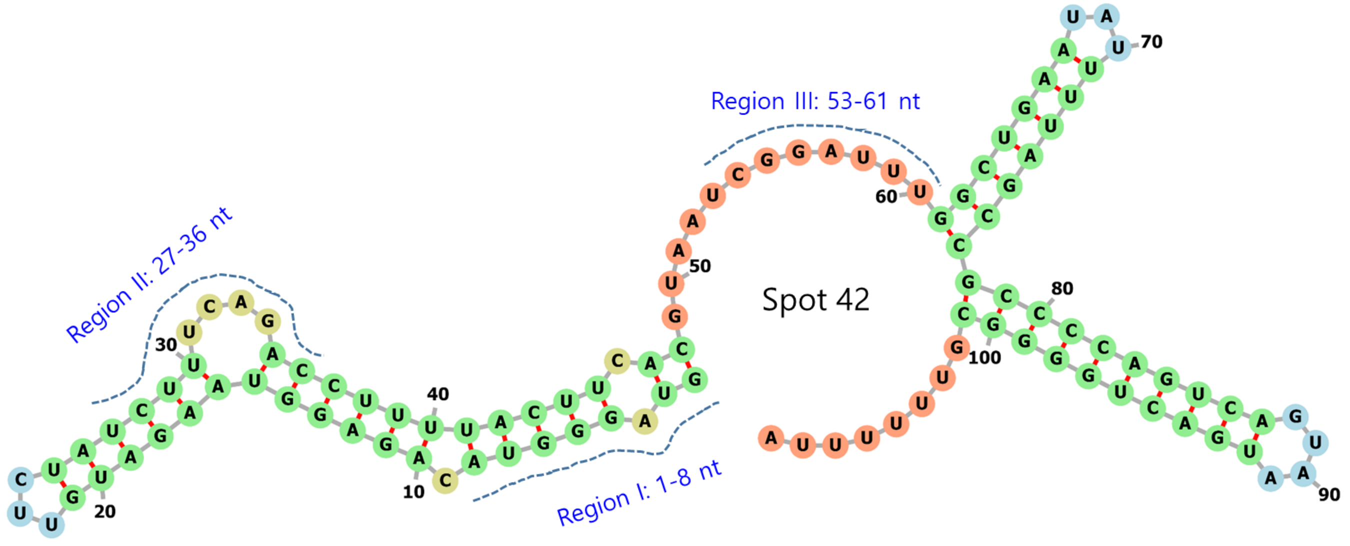

2 Supplementary Table S1. Primers used in this study and usage.

Primer name	Primer sequence 5' to 3'	Usage
E1-F	ATGAGAGTTCTGGTTACCGGTGGTA	Northern E probe generation (500 bp)
E2-R	TGGGCTTTTTGCAGATCGGTGAGGA	
K1-F	ATGAGTCTGAAAGAAAAAACACAAT	Northern K probe generation (501 bp)
K2-R	GCCTACAAACTGGTTTTCTGCTTCC	
DMI-1-F	GACGTCTAGGGTACAGAGGTAAG	Site-directed mutagenesis
DMI-3–F	GACGTCGGGTACAGAGGTAAG	
DMI-5–F	GACGTCGTACAGAGGTAAG	
MMII-1-R	CGTGAAGTAAAAGCTCTGAAAGATAGAAC	
MMII-2-R	CGTGAAGTAAAAGCACTGAAAGATAGAAC	
MMII-3-R	CGTGAAGTAAAAGCAGTGAAAGATAGAAC	
MMIII-1-R	AATATTCAGCCAAATCCGCTTACGTGAAG	
MMIII-3-R	AATATTCAGCCAAAAGGCCTTACGTGAAG	
MMIII-5-R	AATATTCAGCGCTATCCGATTACGTGAAG	
5SF	GAGAGTAGGGAACTGCCA	5′ RACE PCR primer
Spf-23n-R-ext	AAAGATAGAACATCTTACCTCTG	5′ RACE for Spot 42
T2-R	CTGACCGTGCGGATGCGGGTTAGAG	5`RACE PCR primer (*galTKM* mRNA)
K2-R	AGCCTACAAACTGGTTTTCTGCTTCC	5`RACE PCR primer (*galKM* mRNA)
M2-1-R	CATCTGAACTCAGGGCAAACA	5`RACE PCR primer (*galM* mRNA)
ET-ext-R	AGAATCCATTGCCCGGTGAG	5`RACE extension primer (*galTKM* mRNA)
TK-ext-R	ATGGTGTGAGTGGCAGGGTA	5`RACE extension primer (*galKM* mRNA)
KM-ext-R	TGCCAGTGCGGGAGTTTCGT	5`RACE extension primer (*galM* mRNA)
*hfq-Hind*III-F	CATATGATGGCTAAGGGGCAATCTTTA	Construction of pHfq plasmid
*hfq-Hind*III-R	CTCGAGTTATTCGGTTTCTTCGCTGTCC	
QPCR_hfq-77-F	TGGTGAATGGTATTAAGCTGC	Real-time qPCR for detecting *hfq*
QPCR_hfq-184-R	CAGTAGAAATCGCGTGCTTGT	
RT- *rrsB*-for	CAGAATGCCACGGTGAATACGTTCC	Real-time qPCR for detecting *rrsB*
RT- *rrsB*-rev	CAACCCACTCCCATGGTGTGA	

References

1. Datsenko KA, Wanner BL. One-step inactivation of chromosomal genes in *Escherichia coli* K-12 using PCR products. *Proceedings of the National Academy of Sciences of the United States of America*. 2000; 97(12):6640-5.

2. Lee, H.J., Jeon, H.J., Ji, S.C., Yun, S.H., and Lim, H.M. (2008) Establishment of an mRNA gradient depends on the promoter: an investigation of polarity in gene expression. *Journal of molecular biology* 378: 318-327.

3. Wang X*, et al.* (2014) Expression of each cistron in the gal operon can be regulated by transcription termination and generation of a galK-specific mRNA, mK2. *Journal of bacteriology* 196(14):2598-2606.

4. Wang X, Ji SC, Jeon HJ, Lee Y, & Lim HM (2015) Two-level inhibition of galK expression by Spot 42: Degradation of mRNA mK2 and enhanced transcription termination before the *galK* gene. *Proceedings of the National Academy of Sciences of the United States of America* 112(24):7581-7586.

5. Jeon, H.J., Kang, C., N, M.P.A., Lee, Y., Wang, X., Chattoraj, D.K. and Lim, H.M. (2020) Translation Initiation Control of RNase E-Mediated Decay of Polycistronic *gal* mRNA. *Frontiers in molecular biosciences*, 7, 586413.
